# Antifungal Agents in Agriculture: Friends and Foes of Public Health

**DOI:** 10.3390/biom9100521

**Published:** 2019-09-23

**Authors:** Veronica Soares Brauer, Caroline Patini Rezende, Andre Moreira Pessoni, Renato Graciano De Paula, Kanchugarakoppal S. Rangappa, Siddaiah Chandra Nayaka, Vijai Kumar Gupta, Fausto Almeida

**Affiliations:** 1 Department of Biochemistry and Immunology, Ribeirao Preto Medical School, University of Sao Paulo, Ribeirao Preto, SP 14049-900, Brazil; veronica.sbrauer@usp.br (V.S.B.); ca.rezende8@gmail.com (C.P.R.); andrepessoni1@gmail.com (A.M.P.); 2 Department of Physiological Sciences, Health Sciences Centre, Federal University of Espirito Santo, Vitoria, ES 29047-105, Brazil; renato.gracciano@gmail.com; 3 Department of Studies in Chemistry, University of Mysore, Manasagangotri, Mysore 570006, India; rangappaks@gmail.com; 4 Department of Studies in Biotechnology, University of Mysore, Manasagangotri, Mysore 570006, India; moonnayak@gmail.com; 5 Department of Chemistry and Biotechnology, ERA Chair of Green Chemistry, Tallinn University of Technology, 12618 Tallinn, Estonia

**Keywords:** Antifungal agents, fungicides, agriculture, chemoinformatics

## Abstract

Fungal diseases have been underestimated worldwide but constitute a substantial threat to several plant and animal species as well as to public health. The increase in the global population has entailed an increase in the demand for agriculture in recent decades. Accordingly, there has been worldwide pressure to find means to improve the quality and productivity of agricultural crops. Antifungal agents have been widely used as an alternative for managing fungal diseases affecting several crops. However, the unregulated use of antifungals can jeopardize public health. Application of fungicides in agriculture should be under strict regulation to ensure the toxicological safety of commercialized foods. This review discusses the use of antifungals in agriculture worldwide, the need to develop new antifungals, and improvement of regulations regarding antifungal use.

## 1. Introduction

There are >99,000 known species of fungi, including molds, yeasts, mushrooms, and polypores [1,2]. Fungi can grow in almost all habitats, including soil, air, seas, rivers, as well as on organic matter, including food, and other organisms, such as plants, animals, and even human skin [3]. Fungi have several significant impacts on society besides causing diseases. They are used for food production, as well as for pharmaceutical preparation, agricultural purposes, and organic-matter decomposition [4].

Among the thousands of characterized fungal species, only a few hundred are infectious and cause diseases in humans [5]. The immune system of a healthy individual has several effective mechanisms to identify, control, and eliminate fungal infections. However, in pathological conditions, including acquired immunodeficiency syndrome (AIDS), tuberculosis, diabetes mellitus, and cancer, or under increased physiological stress, such as during organ transplantation, corticosteroid administration, and chemotherapy [6,7], the risk of developing fungal infections-related ailments is highly increased. [8] Among the infectious fungal species, *Aspergillus* spp., *Candida* spp., *Cryptococcus* spp., and *Pneumocystis jirovecii* are the causative agents of major mycoses in humans [9,10]. In addition, the incidence of infections caused by *Zygomycetes*, *Fusarium*, and *Scedosporium* has been rising [11,12]. Recent global estimates have revealed approximately 3,000,000, 250,000, 700,000, and 223,100 cases of chronic pulmonary aspergillosis, invasive aspergillosis, invasive candidiasis, and cryptococcal meningitis among AIDS patients [13]. Moreover, the mortality rates associated with invasive fungal infections are >50% even with antifungal treatment, possibly because of the late diagnosis of the infections and identification of the causative fungi [3,11].

It is difficult to develop novel fungicides with ideal characteristics, including broad-spectrum effectiveness, enhanced bioavailability, and minimal toxicity and side effects, due to similarities between fungal and mammalian cells, such as in the biosynthetic pathways and chromatin organization of DNA [6,14]. Consequently, drug development against invasive fungal pathogens has been slow. It began in the 1950s, with the approval of polyene amphotericin B deoxycholate, which was followed by the development of the pyrimidine analog flucocytosine in the 1960s, azoles in the 1970s, and echinocandins in the 2000s [15]. Fungi have plastic genomes and reproduce rapidly [16]. In addition to these properties, the increased usage of prophylactic antifungal agents and empirical and directed therapies has increased the number of drug-resistant pathogenic fungal strains [17]. Moreover, antifungal drugs used in agriculture can serve as environmental drivers for the development of drug-resistant fungal strains [18]. To decrease the development of drug resistance of fungi in fields, the use of a mix of antifungals with different action mechanisms is encouraged [16,17]. Thus, the aim of this review is to discuss the use of antifungal agents in agriculture, its correlation with the development of drug-resistant fungal strains, as well as the consequences of unregulated antifungal use on public health.

## 2. Agricultural Fungicides

**Figure 1:**
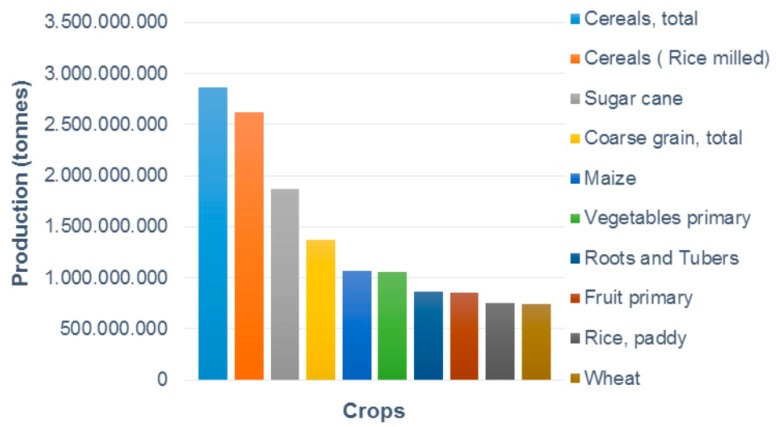
The annual global production of major crops during 2013–2017 (Source: FAOSTAT. www.fao.org. Access on: 06 November 2018).

Agricultural pesticides are chemicals that are used to kill crop pests or inhibit the growth or harmful effects of these organisms [19]. Among the different classes of pesticides, fungicides include physical, chemical, or biological agents intended to combat fungal microorganisms [20]. These are widely used in agricultural systems to control diseases and preserve the yield and quality of crops [21]. The history of the agricultural sector is shaped by constant challenges with respect to increasing productivity and, thus, supply to meet the increasing need for consumption, and, thus, demand [20]. The increased need for productivity has mainly been addressed by the eradication of pests through the use of pesticides [22]. According to the Food and Agriculture Organization of the United Nations (FAOSTAT), the main crops produced in the world between 2013 and 2017 were cereals (especially coarse grains, maize, rice, and wheat), sugarcane, primary vegetables and fruits, and plants grown for their roots and tubers (Figure 1) [23]. During this period, the African continent was the main producer of cereals and roots/tubers, for which the yearly average production was 5 M tons (80.4%) and approximately 7 M tons (71.7%), respectively. Asia’s main agricultural product during the same period was rice, with a yearly average production of 668 M tons, corresponding to 90.4%. China, India, and Indonesia showed the highest agricultural productivity among Asian countries [23]. The American continents had a high production of soybeans (88.2%), sugarcane (54.8%), and maize (50.4%). For instance, on average, the United States alone produced 363 and 108 M tons of maize and soybeans, respectively, per year. Brazil’s main agricultural product during this period was sugarcane, with a yearly average production of 756 M tons. Additionally, it was the second and third largest producer of soybeans and maize, respectively [23].

Fungal phytopathogens affecting agricultural crops lead to a decrease in their quality and production [24]. They act as a threat to crops [25] through various mechanisms of pathogenesis that compromise the immune system of the plants (Table 1) [24]. The use of fungicides against fungal plant diseases improves crop yield, quality, and shelf-life [21,26]. Some examples of antifungal agents include benzimidazoles, dithiocarbamates, strobilurins, and azoles [26], with azoles, especially triazoles, being widely used in fields [27].

The first compound with fungicidal properties was described by Bénédict Prévost in 1807 when he found that germination of spores from *Tilletia caries* was inhibited by pieces of metallic copper placed in the soil [39]. At the beginning of the 20th century, the first organic fungicide, an organomercurial compound, was synthesized. Further studies in this direction resulted in the commercialization of several fungicides, such as 2-methoxyethyl silicate and 2-hydroxyphenyl mercury, which are effective against fungi, such as *Fusarium* spp. and *Dreschlera* spp. [40].

In recent years, the agricultural sector has faced several challenges involving decreased crop yield due to pests, diseases, and abiotic stresses [41]. In addition, the global population is estimated to grow by approximately 30% by 2050, necessitating investments to increase agricultural production and productivity [23]. Therefore, the use of efficient fungicides for protection of agricultural crops from disease during both large-scale agricultural production and post-harvest stages is necessary [42].

**Figure 2:**
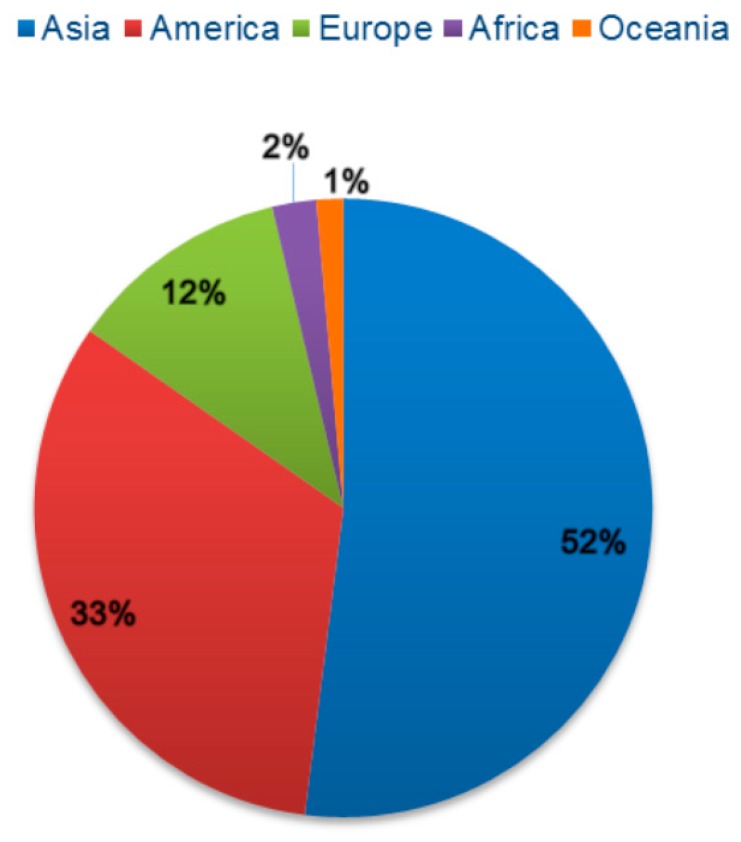
Use of pesticides in the world during 2012–2016 (Source: FAOSTAT. www.fao.org. Access on: 17 December 2018).

The global pesticide use increased significantly during 2012–2016, with a peak observed in 2014. During this period, Asia showed the highest pesticide use, totaling 2 M tons (52%) of average pesticide use, followed by America with an average use of 1 M tons (32.7%), Europe with 477 K tons (11.6%), Africa with 95 K tons (2.3%), and Oceania with approximately 55 K tons (1.4%) (Figure 2). Brazil used the highest amounts of fungicides [23], followed by Italy, Spain, France, Colombia, United States of America, Mexico, Japan, Turkey, and Ukraine [23]. Data from the National Union of the Product Industry for Plant Protection (SINDIVEG) showed that, in 2016, fungicides became the most commercialized product category in Brazil, accounting for 33% of the total market [43].

### 2.1. The Use of Antifungal Agents in Agriculture Poses a Potential Threat to Human Health

Different scientific studies conducted in the late 1950s and early 1960s showed that agricultural pesticides might threaten human health in the long term [44]. These threats derive from the exposure of consumers and workers to pesticides through contact, inhalation, or ingestion of food or water contaminated with pesticides [20]. Adverse effects of such exposures have been reported and include endocrine, immunological, neurological, and carcinogenic problems, as well as premature births [44].

In addition to health problems, the excessive use of pesticides can also cause environmental problems; accumulation of pesticides in the environment disrupts the ecological balance and gives rise to pathogenic resistance to the pesticides [45,46]. Thus, the application of fungicides in agriculture should be under strict regulation to ensure that commercialized foods are safe for consumption and pose negligible risks of acute toxicity due to carry-over [42].

### 2.2. Antifungal Resistance

Antifungal resistance is a heritable fungal characteristic that develops through natural selection of fungi [47]. The selective pressure exerted on fungi by exposure to a fungicide “selects” one or more strains that exhibit resistance to that fungicide or have a “fungicide-resistant phenotype” [48,49]. These strains can, then, survive and reproduce in the presence of this fungicide [48]. Biological factors that enable the spread of a fungicide-resistant fungal pathogen include a short life cycle, abundant sporulation, and long-distance spore dispersal [50]. In addition to these factors, which are intrinsic to the fungal species, development of fungicide resistance depends on how the fungicide is used [51]. There are four major mechanisms of fungal resistance development: (i) alterations in the target protein due to mutations, (ii) upregulation of the target protein, (iii) decrease in drug effective concentration, mainly because of development of efflux processes, and (iv) detoxification by metabolic enzymes, resulting in the degradation of the fungicide [14,21,52]. The possible mechanisms of resistance development against various antifungal drugs are summarized in Table 2 [16]. The most important and frequently used antifungals in agriculture are discussed below.

#### 2.2.1. Methyl Benzimidazole Carbamate (MBC)

MBC or Benzimidazole, is a heterocyclic compound and a benzo derivate of imidazole [73]. Described in 1969, it exhibits several biological activities, such as anti-parasitic, anti-helminthic, anti-viral, and anti-neoplastic activities among others [74]. Since its discovery, benzimidazole has been widely used for crop management [75] as an innovative fungicide with a systemic curative activity that allows for longer intervals between consecutive sprays [65]. This antifungal agent inhibits microtubule assembly, mainly by binding to the free β-tubulin monomers in the colchicine-binding site [53,76]. Suppression of microtubule formation, in turn, impairs cell division and may lead to cell death [75].

Problems regarding resistance to benzimidazole emerged soon after it was introduced into the market. First observed in *Botrytis* spp. in 1971, benzimidazole resistance has been reported in approximately 115 species of fungi to date [77]. The mechanism of resistance to this class of fungicides involves generation of point mutations in the β-tubulin gene [57]. The first report of such a mutation was published in 1992 by Koenraadt et al. who showed a conversion in codon 198 of the *Venturia inaequalis* β-tubulin. This codon encodes alanine in the β-tubulin protein of the ordinary strain, glutamic acid in an MBC-sensitive strain, lysine in a highly MBC-resistant strain, and glycine in a moderately MBC-resistant strain. Another mutation was reported in codon 200 of a medium resistant strain, which generates phenylalanine instead of tyrosine [56]. In addition, mutations in codons 6, 50, 167, and 240, which may lead to benzimidazole resistance, have been reported in field isolates [57].

#### 2.2.2. Succinate Dehydrogenase Inhibitor (SDHI)

Carboxin, a generation I SDHI, was the first fungicide of this class. It was introduced into the market in 1966 [59] and targets basidiomycete pathogens [21]. Generation II SDHIs, including boscalid, fluxapryroxad, and fluxypyram, exhibit high antifungal activity in cereals, fruit trees, vegetables, and field crops [62]. This class of antifungals inhibits fungal respiration by blocking the ubiquinone-binding site (Q-site) in complex II of mitochondria [61].

SDHIs show a broad-spectrum activity, but their intensive application likely causes selective pressure leading to the development of resistant pathogen strains [59]. Since this class of fungicides includes single-site inhibitors, the FRAC (Fungicide Resistance Action Committee) classified them as posing medium–high risk for emergence of resistant strains [65]. Interestingly, there are reports of an association between carboxin and boscalid resistance and mutations in the succinate dehydrogenase gene [62]. The most common mutations involved are the substitutions H257L and H257Y, which replace the histidine residue at position 257 (a ubiquinone-binding site) of the succinate dehydrogenase gene with leucine and tyrosine, respectively [58,62]. *Botrytis cinerea*, *Alternaria alternate*, *Didymella brioniae*, *Podosphaeera xanthii*, and *Corynespora cassiicola* are examples of fungi that have been reported to be resistant to SHDI [62].

#### 2.2.3. Anilinopyrimidine (AP)

Anilinopyrimidine (AP) was introduced into the market between 1992 and 1995 and is used against ascomycetes [68]. Cyprodinil, mepanipyrim, and pyrimethanil belong to this fungicide group [65]. This class of fungicides is used to control gray mold caused by *B. cinerea* in fruits, vegetables, and ornamental flowers and also to control apple scab caused by *Venturia inaequalis* [68].

The action mechanism of AP involves the inhibition of methionine synthesis and hydrolytic enzyme secretion (proteases, cellulases, cutinases, and lipases) [64]. It is considered to pose a moderate risk of resistance development [65]. AP resistance has been reported in *B. cinerea*, *V. inaequalis*, and *Oculimacula* spp. [51]. Among these, AP-resistant *B. cinerea* strains have been reported in several European vineyards [55]. Low concentrations of AP were used to inhibit the tube-elongation and mycelial growth of wild-type *B. cinerea* strains. However, the AP-resistant *B. cinerea* strains Ani R1, Ani R2, and Ani R3 have emerged with time. These strains exhibit a significant degree of resistance to AP, with Ani R1 showing moderate to high resistance at all stages and Ani R2 and Ani R3 showing resistance only in the germ-tube elongation stage [55]. The possible mechanism through which strains Ani R2 and Ani R3 resist AP activity might be the energy-dependent efflux (ABC-transporter) of the fungicide [69]. Mutations at the target sites of AP in Ani R1 have been reported [69]; however, more studies are needed to identify the molecular mechanisms underlying AP-resistance in this strain [68,69].

#### 2.2.4. Qo Inhibitor (QoI)

QoI is also called “strobilurin” because this class of fungicides was derived from a natural compound called strobilurin A produced by mushrooms (basidiomycetes) of the genera *Strobilurus* [78,79]. This class of natural compounds is unstable in the presence of light and, thus, is not useful for the management of crop diseases [79]. However, modifications introduced into the chemical structure of one of these compounds generated a photo-stable version with antifungal activity, allowing strobilurin to be introduced into the market in 1996 [78,79]. Currently, there are 18 fungicides of this class, with different chemical groups in their structure, available on the market, including methoxyacrylates, methoxyacetamide, methoxycarbamates, oximinoacetates, oximinoacetamides, axazolidinedones, dihydrodioxazines, imidazolinones, and bezylcarbamates [51,65], but they all share a common mechanism of action.

The name “QoI” arose because this fungicide class inhibits binding at the Qo (quinol oxidation) site of complex III (cytochrome bc_1_ enzyme complex) during mitochondrial respiration [80]. Thus, the electron transfer between cytochrome b and c does not occur, blocking NADPH (nicotinamide adenine dinucleotide) oxidation and ATP (adenosine triphosphate) production [79,81]. QoIs exhibit fast action since the lack of energy in fungal cells affects the spore germination process and zoospore motility [80]. This class has a broad-spectrum activity against fungi, including ascomycetes, basidiomycetes, and oomycetes [65], and is used for treatment of several crops infected with these fungi. Despite these features, QoI is classified by FRAC as a fungicide class with high risk for the development of fungal resistance [51]. The main mechanism of fungal resistance related to this class involves point mutations in the mitochondrial cytochrome b (*cyt b*) gene [57] that result in changes in the amino acid sequence of the protein, preventing fungicide binding to it [80]. The following three-point mutations have been described as the cause of development of resistant phenotypes: substitution of alanine for glycine at position 143 (G143A), leucine for phenylalanine at position 129 (F129L), and arginine for glycine at position 137 (G137R) [70]. These three mutations lead to different degrees of resistance against QoIs; G143A is associated with high resistance and F129L and G137R are associated with moderate resistance [70]. At least 20 pathogens have been reported to have resistance against QoIs around the world [82]. Among the described QoI-resistant fungi, *Erysiphe necator*, *Pseudopernospora cubensis,* and *V. inaequalis* carry the G143A mutation [83], whereas *Alternaria solani*, *Pyrenophora teres*, and *Pythium aphanidermatum* carry the F129L mutation, and *Pyrenophora tritici-repentis* carries the G137R mutation [65].

#### 2.2.5. Morpholine

Morpholine is an organic compound with a heterocyclic ring containing oxygen and nitrogen and has various biological effects, such as anti-parasitic, anti-cancer, anti-inflammatory, anti-malarial, and anti-fungal effects [84]. Its antifungal action was described in 1965, and dodemorph, tridemorph, aldimorph, and fenpropimorph are some of the members of this antifungal class [65].

Morpholines are systemic fungicides used to control powdery mildews and cereal foliar diseases [85]. The class exerts its antifungal activity via the inhibition of two enzymes involved in ergosterol synthesis: ∆14-reductase and ∆8-∆7-isomerase [6]. The FRAC classifies morpholines as posing a moderate risk for resistance development [51]. Although there have been reports of decreased sensitivity to this class of fungicides in powdery mildews, the mechanism underlying this resistance remains unknown [16].

### 2.3. Azole Resistance of Aspergillus: Implications in Clinic and Fields

Alterations in target proteins have been demonstrated for several fungicides, including azoles, the main antifungal class used for crops [16]. Azoles are generally sprayed in fields to control rust and mildew affecting fruits, vegetables, cereal, and other crops [86,87]. Azoles have a synthetic origin and a cyclic structure like imidazoles and triazoles [12]. Their mechanism of action involves interfering with the enzymatic activity of lanosterol 14α-demethylase (also known as CYP51), a member of the P450 enzyme family. This enzyme converts lanosterol to ergosterol, which is an essential component of the fungal cell membrane and contributes to its fluidity and integrity as well as the efficient functioning of membrane-bound enzymes. Inhibition of CYP51 activity results in the accumulation of demethylated lanosterol at toxic rates, disturbing the dynamics and stability of the cell wall. Consequently, the fungal growth and replication become suppressed [12,26,27,88]. Azoles are extensively used, since they are inexpensive, have a broad spectrum action, and are effective against plant fungal diseases. In addition, these fungicides are used in grain and grass environments during pre- and post-harvest periods to prevent contamination by yeast (such as *Candida* spp., *Thrichosporon penicillatum*, and *Cryptococcus* spp.) and filamentous fungi (such as *Aspergillus* spp., *Fusarium* spp., and *Alternaria* spp.) [87]. However, the excessive use of azoles leads to contamination of soil, air, and plants, mainly because of their lipophilic characteristic, which results in their absorption into soil and organic matter. Azoles exhibit high stability, and can remain virtually unchanged in the environment and in food for months [87,89].

Fungal resistance to azoles could be due to various factors. Mutations in *cyp51A* can reduce the affinity of the encoded protein to its inhibitors and upregulation of this gene increases azole efflux by upregulation of membrane transporters [72]. In field isolates, the most common reason for azole-resistance has been found to be mutations in *cyp51A* [71,72]. *Zymoseptoria tritici* harbors >30 modifications in *cyp51A* [71]. Species, such as *V. inaequalis*, *Penicillium digitatum*, *Cercospora beticola*, *Monilinia fructicola*, and *Blumeriella jaapii*, are associated with azole-resistance caused by *cyp51A* upregulation resulting from insertions of variable sizes in the gene promoter [72]. Efflux transporters, such as ATP-binding cassette (ABC) transporters and major facilitator superfamily (MFS) transporters, can reduce the azole concentration in fungal cells. Among the field isolates of fungi, this type of resistance has been reported for *B. cinerea*, *Penicillium digitatum*, and *Zymoseptoria tritici* [71].

The extensive use of azoles in agriculture can affect phytopathogens with medical relevance [26]. Consequently, fungi causing important human mycoses may also develop azole-resistance [26,87]. Several human diseases are caused by fungi that survive in various environments and foods, such as *Coccidioides*, *Histoplasma*, *Aspergillus*, and *Cryptococcus* [87]. *Aspergillus fumigatus*, a saprophytic fungus that can live in soil, produces spores that are airborne and can be inhaled by humans. The acquired resistance of *A. fumigatus* against commonly used antifungal drugs might be due to the extensive use of fungicides [90,91]. In the clinic, *A. fumigatus* resistance has been observed in patients who received long-term azole therapy against aspergillosis, mainly because azoles are the first choice of drugs used for the treatment of this fungal disease [92]. However, several cases of *A. fumigatus* resistance occurred in patients who were never treated with azoles; thus, it has been hypothesized that there could be other sources of exposure to fungi with acquired resistance against azoles, such as agricultural crops [93].

Snelders et al. (2008) reported such an association between *A. fumigatus* azole resistance and environmental exposure [94]. They investigated the prevalence of intraconazole resistance in 1912 clinical isolates of *A. fumigatus* collected from 1219 patients at the University Medical Centre in Nijmegen during a period of 12 years and compared them with the clinical isolates from hospitals in different cities. They confirmed that there was a wide-spread azole resistance among the intraconazole-treated samples, with 94% of the resistant isolates carrying *cyp51A* mutations. Since person-to-person transmission of *Aspergillus* infection is not common, a similar TR_34_/L98H mutation in unrelated patients indicated the involvement of environmental factors. For instance, it is possible that the modified conidia were dispersed by the air and, consequently, caused the infection [94].

The main mechanism underlying azole resistance acquired by *A. fumigatus* involves point mutations in or upregulation of *cyp51A* (14α-demethylase in *A. fumigatus*) [95]. Several point mutations in *cyp51a*, such as G54, M220, G448S, G138, P216, and F219, have been reported to confer azole resistance onto *A. fumigatus* [93,96]. In addition, polymorphisms that lead to amino acid mutations, such as F46Y, M172V, N248T, D255E, and E427K, have already been associated with the azole resistance of *A. fumigatus* [95]. Tandem insertions (46-bp long in total) in the *cyp51a* promoter region and substitution of tyrosine 121 to phenylalanine and threonine 289 to alanine (TR_46_/Y121F/T289A) were found to be associated with voriconazole resistance. In addition, a 53-base pair sequence inserted in tandem in the promoter region has been correlated with resistance to azoles. The mutation TR_34_/L98H, which results in the overexpression of *cyp51A*, is the main mutation associated with *A. fumigatus* resistance to azoles [94,95]. Azole resistance among clinical isolates of *Aspergillus fumigatus* has been demonstrated in a recent study [97]. In this report, three isolates with itraconazole resistance carried diverse *cyp51A* mutations. One of these isolates harbored the mutation M220K, while a second exhibited the G54 mutation in addition to a modification in the *cyp51A* promoter. The third isolate had an integration of a 34-bp tandem repeat (TR34) in the promoter region of the gene and an L98H substitution (substitution of leucine 98 with histidine).

The phenomenon of azole-resistance of *A. fumigatus* is widespread and it has been reported in Middle East, Asia, Africa, Australia, North Europe, and South America [98]. It is not yet clear where or when this mechanism of resistance initiated; however, it has been suggested that it is probably due to a common ancestral gene, since there is lower genetic diversity among unrelated *A. fumigatus* strains [98]. It is important to highlight that approximately 70% of patients with azole-resistant aspergillosis had not undergone any azole treatment at all [98,99]. Meireles et al. [100] evaluated the change in the clinical antifungal sensitivity of *Aspergillus flavus* in response to azole and benzimidazole fungicides [100]. They showed that exposing *Aspergillus flavus* to azoles changed the sensitivity of the fungus to the antifungals itraconazole, voriconazole, and posaconazole, evidencing the development of resistant phenotypes, and constituting the first case report of antifungal resistance induced by azole exposure. Dos Reis et al. [101] investigated the *Aspergillus fumigatus MSH2* mismatch repair (MMR) gene *mshA* and its impact on virulence and evolution of azole resistance [101]. The *mshA* mutant *A. fumigatus* strain showed significantly reduced virulence in a neutropenic murine model of invasive pulmonary aspergillosis. In addition, the mutant strain exhibited a rapid acquisition of virulence and high levels of resistance to posaconazole.

In this context, public health surveillance programs for fungal diseases must be put in practice because the excessive use of azole in the field is already causing harm in clinical settings [94]. Azoles are the first line of treatment against *Aspergillus* infection. As diseases caused by this fungal species, including invasive aspergillosis, allergic manifestations, and chronic pulmonary disease, can affect a large number of people, fungal acquisition of azole resistance will lead to increased mortality due to *Aspergillus* infections around the world [17,102].

## 3. New Antifungal Strategies

Chemical control is essential for the maintenance of reliable and good crop yields [103]. To conserve fungicides available in the market and to protect the new arrivals, further research along with increased cooperation between industries and regulatory agencies is required [51]. However, the search for new fungicides has been challenging due to increased resistance to fungal pathogens [104]. Awareness regarding environmental safety has generated public demand for effective and safe antifungal biocontrol agents that are economically, environmentally, and socially sustainable [105]. Nanotechnology has emerged as a new research area in the present century and allows the use of nanoparticles and nanomaterials for protection of agricultural crops against fungal pathogens [106]. Copper nanoparticles have received increasing attention in this regard because their antimicrobial activity has been known since ancient times [107,108]. Copper was used in agriculture in 1761 for the first time, when it was observed that soaking seed grains in a weak solution of copper sulfate inhibited the growth of fungi present in the seeds [109]. Since then, copper compounds have been widely used in agricultural practices, such as for antifungal [110] or antimicrobial purposes [111,112].

Kanhed et al. showed that copper nanoparticles exhibit promising antifungal activity against phytopathogenic fungi. They also observed that these nanoparticles exhibited better antifungal activity than that of bavistin, which is a commercially used fungicide. Therefore, copper nanoparticles might be used in agriculture as a novel antifungal strategy for the control of fungal pathogens of plants [106].

Besides metallic nanoparticles, chitosan nanoparticles have been proposed as potential biopesticides against fungal infections [113]. The antifungal activity of nanoparticles in agriculture is poorly studied; however, some studies have focused on the use of low concentrations of nanoparticles with low toxicity in the agricultural sector [114]. Servin et al. (2015), and Ditta and Arshad (2016) demonstrated that nanomaterials can suppress plant diseases, increase agricultural yield, and provide more nutrients to plants than fertilizers [115,116].

Several plants have been used for therapeutic and prophylactic treatment against several infectious diseases since ancient times. Some good, natural-based fungicides have showed promising antifungal activity and should be a good alternative to combat fungal pathogens in agriculture. Volatile constituents from *Origanum onites* have been evaluated regarding to their antifungal activity against the pathogens *Phomopsis obscurans*, *Fusarium oxysporum*, *Colletotrichum species*, and *Botrytis cinerea* [117]. Thus, a better understanding of the natural based fungicide strategies and its impact on plant-fungal interactions could decrease the use of pesticides in agriculture.

Recently, Hao et al. (2017) analyzed the antifungal activities of ferric oxide (Fe_2_O_3_), copper oxide (CuO), and titanium oxide (TiO_2_) nanoparticles and three carbon nanomaterials, multi-walled carbon nanotubes (MWCNTs), fullerene (C60), and reduced graphene oxide (rGO), against the fungus *B. cinerea* [114]. This fungus damages fruits, vegetables, and ornamental plants [118]. In plants, such as roses, it causes gray mold, which results in a loss of approximately 30% plant productivity per year [119]. Through in vitro and in vivo experiments, Hao et al. (2017) found that each of the three aforementioned carbon nanomaterials inhibited *B. cinerea* infection in roses at concentrations of 200 mg/L. This inhibitory effect derives from the interaction between the carbon nanomaterials and fungal spores, leading to the aggregation of the spores and, thus, suppressing their germination. As with the Fe_2_O_3_ and CuO nanoparticles, carbon nanomaterials showed significant antifungal effects even at concentrations of 50 mg/L. While TiO_2_ nanoparticles exhibited no evident effects, there was a decrease in the number of micelles formed by them relative to the control. Therefore, this study demonstrated the antifungal activity of carbon nanomaterials and metal nanoparticles in *B. cinerea* and emphasized that the application of nanoparticles should be carefully evaluated because of their potential toxicity and environmental risks [114].

To date, various strategies have been developed to reduce antifungal resistance in agricultural settings while promoting the development of new antifungal agents [16]. Currently, combinatorial fungicide treatment constitutes one of the strategies used to delay antifungal resistance [120,121]. In addition, research in the areas of synthetic biology and epigenomics has allowed the development of new antifungal agents based on RNA interference approaches [122], such as bi-directional trafficking of plant-fungal miRNAs, for the control of pathogens, including *B. cinerea* [123]. Such approaches for the development of new antifungal strategies are promising and potentially transformative [16].

### 3.1. Endophytic Fungi in Agriculture

In recent decades, the search for alternatives for disease control in agriculture has been gaining prominence because some fungal pathogens have gained antifungal resistance and some synthetic chemicals have been banned for being pollutive and toxic [124]. Recent advances have shown that the use of microorganisms, mainly bacteria and fungi, as biological control agents has been advantageous for the control of diseases and pests, improving agricultural yields [125,126].

Endophytic fungi have been studied as an alternative and sustainable means of converting the natural compounds in host plants to antifungal compounds, which are not only effective against human pathogens but also against phytopathogens [127]. Endophytic microorganisms are found in plant species of extreme importance [124] and can be classified as competent, optional, obligatory, opportunistic, or passive, depending on their effects on plants [128,129,130,131]. Recently, they have gained special attention due to the benefits these microorganisms can confer onto their hosts in the form of pesticides, helping the growth and survival of plants and increasing their tolerance to extreme temperatures and drought as well as removing contaminants from the soil [132]. *Trichoderma* spp. are associated with the soil and include some important species, such as *T. hamatum*, *T. harzianum*, *T. polysporum*, and *T. viride*, which are important fungal biocontrol agents in plants [133,134]. Many species of *Trichoderma* are used to combat soil fungal pathogens and some follicular pathogens [135,136]. The principal advantages of this type of biocontrol agents are that they easily adapt to various environmental conditions, exhibit tolerance to certain fungicides, have diverse mechanisms of action and simple nutritional requirements, and grow fast [124,137].

Another beneficial approach involving endophytic fungi makes use of their production of bioactive secondary metabolites, which represent a group of microorganisms capable of synthesizing new compounds [138] that can target plant pathogens and pests [139]. Deshmukh et al. (2018) reported several metabolites produced by endophytic fungi from medicinal plants and their potential as antifungal agents [127]. For example, scleroderma A and B and triterpenoid lanostane are produced by an endophyte basidiomycete fungus associated with the *Eucalyptus grandis* plant and these compounds were identified as potential fungicides against *Candida albicans*, *C. tropicalis*, *C. crusei*, and *C. parapsiosis*. Scleroderma B showed better antifungal activity against all these fungal species than scleroderma A or triterpenoid lanostane [140].

*Xylaria* spp., associated with the *Azadirachta indica* plant from China, are a source of new bioactive compounds, some of which exhibit relevant pharmacological properties for drug discovery [141], presenting with antifungal activities against *C. albicans*, *Aspergillus niger*, and *Fusarium avenaceum* [127,142]. The fungus *Mycosphaerella*, which is an endophyte of the plant *Eugenia bimarginata* from Brazil, has been shown to produce two eicosanoid acids that have antifungal activities against *C. neoformans* and *C. gattii* [143]. The cryptocandin lipopeptide is isolated from the fungus *Cryptosporiopsis quercina*, which grows in wood species in Europe. This compound is active against several fungi pathogenic against plants, including the fungal species *B. cinerea* [144], and is related to some antimycotic compounds, such as echinocandins and pneumocandins (Table 3) [145].

Novel molecular biology approaches have been used for the identification and characterization of genetic elements and metabolites involved in the interactions between plants and endophytic microorganisms [146]. For these endophytic microorganisms to be marketed as successful biocontrol agents, certain criteria must be met regarding their ease of application and dissemination to crops without causing any off-target effects. Such microbes present with a wide range of effective modes of action and, under no circumstance, cause symptoms or any adverse effects in their hosts [124]. Thus, this field of research must be further explored as there are still many endophytic microorganisms to be discovered and characterized [124].

### 3.2. Chemoinformatics Approaches for Obtaining New Fungicides

Metabolomics approaches allow the characterization of the metabolites of an organism at a certain time [147]. It aims to identify low molecular weight chemical compounds in biological systems and, combined with other multi-omics technologies, it can be used to investigate and characterize microbial interactions [148]. The most commonly used techniques for metabolism research and structural elucidation of compounds in microbial metabolomics are nuclear magnetic resonance (NMR) and mass spectrometry (MS), in addition to MS separation techniques, such as gas chromatography–MS (GC-MS), liquid chromatography–MS (LC-MS), and capillary electrophoresis–MS (CE-MS) [148,149].

NMR techniques are fast and simple, can be high-throughput, and require a minimal sample amount. However, the main limitation of these techniques is their low sensitivity (micromolar to nanomolar range) [150,151] and high cost. Alternatively, MS platforms allow high-throughput accurate mass determination and structural elucidation, providing higher sensitivity (femtomolar to attomolar range) when applied together with separation techniques, and can lower the costs, depending on the resolution degree required [148,151]. Due to the vast number of compounds produced and complexity of the metabolism, a single method is unlikely to generate a metabolite profile, but the use of multiple analytical instruments and methods can achieve higher coverage, identify biomarkers, and evaluate drug toxicity, efficacy, and selectivity against various pathogenic fungi [150,152].

Alongside these technologies, computational algorithms were developed to extract data from spectral noise obtained, perform statistical analysis, and identify the pursued compounds. Furthermore, the processed data can be overlaid with metabolic pathways and modeled to predict the outcomes of biological experiments, although they might be computationally demanding [148,149]. These very recent techniques allow the identification of changes in response to stimuli and of novel metabolites with potential antimicrobial activities. Their modulation could lead to a unique tool useful for designing drugs aimed at reducing pesticide usage, while preserving crop productivity [153,154]. When the development of such natural bioactive metabolites falls short for the generation of new antimicrobial agents, other approaches should be applied, such as rational design. In general, rational designs use existing knowledge regarding a molecule’s structure or a reaction of interest, combining computational tools and structural knowledge [155]. There are two main chemoinformatics approaches for obtaining new fungicides or other bioactive compounds: structure-based and ligand-based drug design [156]. The first one relies on the knowledge about the three-dimensional structure or the establishment of homology models based on biological receptors, whereas the latter relies on the knowledge about other molecules that bind to the target receptor [156,157].

**Figure 3:**
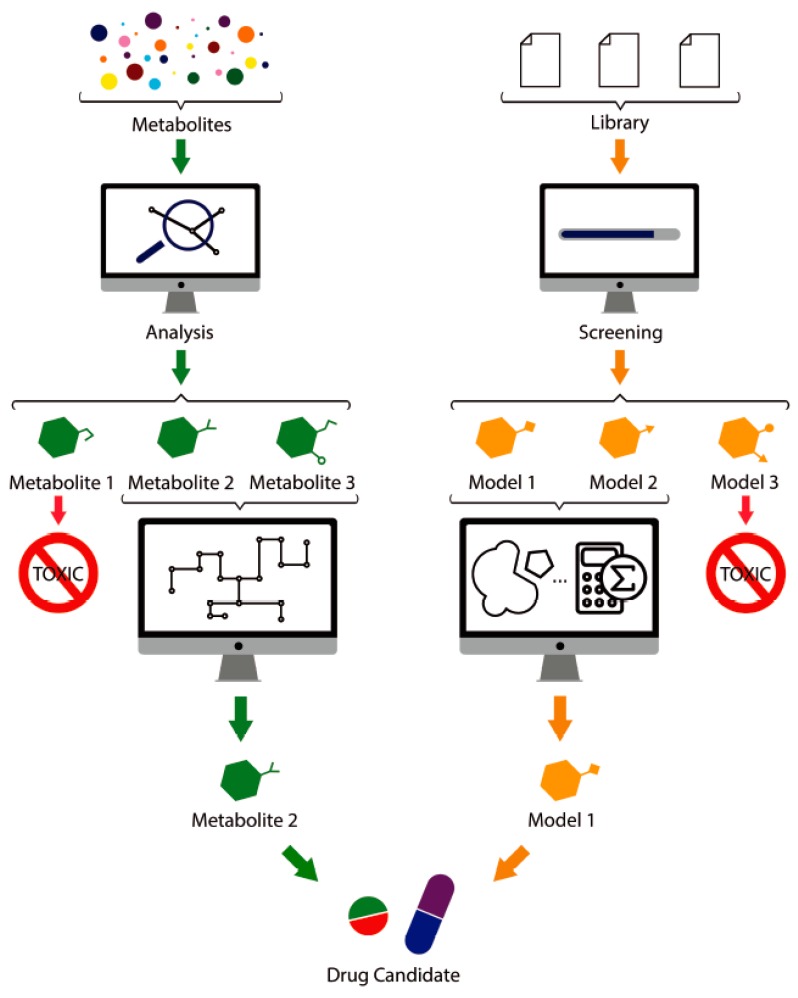
The use of metagenomics in the development of new drug candidates. The left path illustrates metabolite evaluation to identify bioactive or toxic molecules overlaid with metabolic pathways and the selected drug candidate. The right path illustrates the molecular library screening used to generate computational models and calculation of the docking or free energy, leading to the production of a drug candidate.

The high-throughput screening of molecule libraries has advanced drug discovery. This screening relies on quantitative structure–activity relationships (QSAR) and quantitative structure–property relationships (QSPR). Both techniques generate computational models that can predict the biological activity and other properties of a drug based on the molecular structure of a target compound. These models reduce the failure rate of drug targeting, eliminating compounds with previously predicted toxicity or poor pharmacokinetic parameters, optimizing the investigation, and reducing related costs. However, the models are difficult to obtain, and they need to be complemented with the use of other methods, such as molecular modeling, pattern recognition, machine learning or artificial intelligence [158]. In addition, molecular dynamics simulation is also a versatile computational technique to study biological molecules, highly contributing to the development of a rational design at multiple levels. Its combination with other techniques, such as calculation of free energy of molecular docking and binding are essential to elucidate the ligand–receptor interactions, thereby directing a rational investigation [159]. Figure 3 illustrates the schematic process to obtain new drug candidates.

The use of “omics” techniques and other computational methods, integrated with experimental methods, have allowed the search, prediction, and suggestion of new bioactive molecules and drug candidates, such as agrochemical fungicides, saving time and resources and, thus, becoming an alternative for reducing crop losses and overcoming the problem of antifungal drug resistance [156,160,161].

## 4. Conclusions

The use of antifungals in agriculture has increased in recent years. Moreover, the recent rate of emergence of fungicide-resistant pathogenic fungi has restricted the number of commonly used antifungal agents. This has led to the need to develop new antifungal agents. However, research to identify and characterize new antifungal drugs is challenging, and the discovery rate of new molecules with antimicrobial potential is less than the emergence rate of new antifungal-resistant strains. Recently, considerable progress has been made in this field, and the use of nanotechnology has become a promising strategy for the identification of novel antifungal compounds.

## Figures and Tables

**Table 1:** Some important fungal pathogens and their associated diseases in agricultural crops.

Fungal Pathogen	Crops	Disease	Crop Loss (%)
*Botrytis cinerea*	Fruits and ornamental flowers [28]	Gray mold [28]	Up to 30% to 40% loss of strawberries [29]
*Blumeria graminis*	Wheat and barley [28]	Mildews of grasses [28]	18% potential and 13% *de facto* loss of grains under current disease control [30]
*Colletotrichum* spp.	Fruits and vegetables [28]	Anthracnose spots and blights [28]	Losses >80% in tropical, sub-tropical and Mediterranean regions [31]
*Cladosporium fulvum*	Tomato [32]	Tomato leaf mold [32]	Loss of 10–25% during regular years [33]
*Fusarium* spp.	Potato [34]	Dry rot of tubers [34]	Crop losses of up to 25%. During storage, >60% of tubers can be infected [35]
*Fusarium graminearum*	Cereals [28]	Fusarium head blight; Fusarium ear blight or head scab [28]	In China, 5–10% loss. In Europe and South America, up to 50–60% and 70% of loss [30]
*Magnaporthe oryzae*	Rice [28]	Rice blast [28]	Losses vary between 10% and 35% depending on the variety and environmental conditions [25]
*Mycosphaerella graminicola*	Wheat [28]	Septoria tritici blotch [28]	Up to 30–50% loss [25]
*Puccinia* spp.		Rust [28]	70% loss [36]
*Phakopsora pachyrhizi*	Soybean [36]	Rust [36]	Up to 70% loss [36]
*Pytophtora infestans*	Potato [34]	Late blight [34]	16% loss [34]
*Rhizoctonia solani*		Stem canker and black scurf [34]	30% loss [34]
*Sporisorium scitamineum*	Sugarcane [37]	Sugarcane smut [37]	Up to 62% loss [38]
*Ustilago maydis*	Corn [28]	Corn smut [28]	Up to 20% loss [36]

**Table 2:** The main classes of antifungal drugs and the mechanisms of development of resistance to them.

Antifungal Class	Mechanism of Action	Examples of Antifungal Drugs	Examples of Resistant Fungal Species	Mechanism of Resistance
Methyl benzimidazole carbamate	Inhibits microtubule assembly [53]	Benomyl, carbendazim, flubendazole [54]	*Botrytis cinerea*, *Venturia inaequalis* [55,56]	Point mutation in β-tubulin gene [56,57]
Succinate dehydrogenase inhibitor	Inhibition of fungal respiration by binding to the ubiquinone-binding site in the complex II of mitochondria [58]	Carboxin, benodanil, flutolanil, fenfuran, fluxapryroxad, fluxypyram, thifluzamide, furametpyr [59]	*Botrytis cinerea*, *Alternaria alternate*, *Didymella brioniae*, *Podosphaeera xanthii*, *Corynespora cassiicola* [60,61,62,63]	Mutations in succinate dehydrogenase gene (amino acid substitution H257L or H257Y) [58,62]
Anilinopyrimidine	Inhibition of methionine synthesis and secretion of hydrolytic enzymes [64]	Cyprodinil, mepanipyrim and pyrimethanil [65]	*Botrytis cinerea*, *Venturia inaequalis*, *Oculimacula* spp. [66,67,68]	This mechanism is not completely clear; it has been suggested to involve the overproduction of ABC (ATP-binding cassette) transporters or the modification of the target sites [69]
Qo inhibitor	Blocks fungal energy production through inhibition of mitochondrial respiration by binding to the Qo site of complex III [70]	Azoxystrobin, mandestrobin, pyraclostrobin, kresoxim-methyl, dimoxystrobin, famoxadone, fluoxastrobin, fenamidone, pyribencarb [65]	*Erysiphe necator*, *Pseudopernospora cubensis*, *Venturia inaequalis*, *Alternaria solani*, *Pyrenophora teres*, *Pythium aphanidermatum*, *Pyrenophora tritici-repentis* [65]	Point mutations in the mitochondrial cytochrome b (*cyt b*) gene (G143A, F129L, G137R) [57,70]
Morpholine	Inhibition of ergosterol synthesis by blocking ∆14-reductase and ∆8-∆7-isomerase [6]	Aldomorph, fenpropimorph, dodemorph, tridemorph [65]	Decreased sensitivity in powdery mildews [16]	Unknown [16]
Azole	Suppression of ergosterol synthesis by inhibiting 14α-demethylase [12]	Imazalil, oxpoconazole, triflumizole, diniconazole, epoxiconazole, flutriafol [65]	*Zymoseptoria tritici*, *Venturia inaequalis*, *Penicillium digitatum*, *Cercospora beticola*, *Monilinia fructicola*, *Brumeriela jaapii*, *Botrytis cinérea*, *Penicillium digitatum*, *Zymoseptoria tritici* [71,72]	Mutations in *cyp51,* upregulation of *cyp51* and the genes encoding membrane transporters [72]

**Table 3:** Production of secondary metabolites by endophytic fungi and their potential as antifungal agents.

Endophytic Fungi	Plant	Metabolites	Antifungal Activities
Basidiomycete fungus [140]	*Eucalyptus grandis* [140]	Scleroderma A and B Triterpenoid lanostane [140]	*Candida albicans* *C. tropicalis* *C. grusei* *C. parapsiosis* [140]
*Xylaria* spp. [141]	*Azadirachta indica* [127,142]	Bioactive compounds [141]	*C. albicans* *Aspergillus niger* *Fusarium avenaceum* [127,142]
*Mycosphaerella* spp. [143]	*Eugenia bimarginata* [143]	Eicosanoid acids [143]	*Cryptococcus neoformans* *C. gattii* [143]
*Cryptosporiopsis quercina* [144]	Wood species in Europe [144]	Cryptocandin lipopeptide [144]	*Botrytis cinerea* [144]
